# Hepatitis C Seroconversion Remains High among Patients with Regular Hemodialysis: Study of Associated Risk Factors

**DOI:** 10.1155/2022/8109977

**Published:** 2022-12-29

**Authors:** Ni Wayan Wina Dharmesti, I Dewa Nyoman Wibawa, Yenny Kandarini

**Affiliations:** ^1^Division of Gastroentero-Hepatology, Internal Medicine Department, Faculty of Medicine Udayana University/Sanglah Hospital, Denpasar, Bali, Indonesia; ^2^Division of Nephrology, Internal Medicine Department, Faculty of Medicine Udayana University/Sanglah Hospital, Denpasar, Bali, Indonesia

## Abstract

**Methods:**

An analytical cross-sectional study involving patients from 2 dialysis units (1 referral hospital and 1 private dialysis unit) in Denpasar, Bali, Indonesia, from January 2020 to December 2021. We evaluated age, gender, duration of hemodialysis, vascular access, history of transfusion, history of surgery, diabetes mellitus, hepatitis B, human immunodeficiency virus (HIV) infection, and type of dialyzer as possible risk factors of hepatitis C seroconversion among hemodialysis patients.

**Results:**

A total of 338 hemodialysis patients were enrolled in this study. We found hepatitis C seroconversion in 94 patients (27.8%), all of which occurred after regular dialysis was started. Vascular access type (OR 42.07, 95% CI 5.757–307.472) and dialyzer reuse (OR 8.324, 95% CI 4.319–16.044) were showing a statistically significant association with hepatitis C seroconversion. A separate analysis on each dialysis unit found common evidence that the duration of dialysis was significantly associated with hepatitis C infection among hemodialysis patients.

**Conclusion:**

Hepatitis C seroconversion among dialysis patients remains high. Factors related to the dialysis procedure itself played a major role in transmitting the virus.

## 1. Introduction

Hemodialysis patients have a higher risk of HCV infection compared to the general population. The features of the hemodialysis procedure itself may facilitate virus transmissions, such as the risk of blood contamination on devices' surfaces and a large number of patients undergoing dialysis in one shared space [1, 2]. HCV infection in dialysis patients is associated with decreased quality of life and increased all-cause and cardiovascular mortality [2–4]. The great concern for hepatitis C infection among hemodialysis patients is also derived from the evidence of the worldwide increased rate of chronic kidney disease patients requiring renal replacement therapy, where hemodialysis is the predominant modality used in most countries [5].

The prevalence of HCV infection among hemodialysis patients varies greatly across different geographic regions, from a 7.8% seropositivity rate in the United States to a 34.8% prevalence rate reported in Egypt [6–8]. Previous studies in European countries reported decreased prevalence and incidence of HCV infection in patients undergoing hemodialysis [9–11]. However, local HCV outbreaks in hemodialysis units continued to be reported in both Western and developing countries [2]. Data from the Indonesian Renal Registry Report in 2018 have estimated that the national prevalence rates of hepatitis C seropositivity among new and active hemodialysis patients were 8% and 19%, respectively [12]. These figures were higher than the reported national prevalence of hepatitis C in the general population (1%) and were projected to increase annually [12, 13]. Hemodialysis is the most common dialysis modality used in Bali and there has been an increasing demand for hemodialysis treatment. As reported in 2017, there were 1928 new patients (498 per million population) undergoing hemodialysis in Bali compared to 1258 new patients in 2014 [14]. The increased demand was estimated to steeply increase in the future owing to the increased incidence of hypertension and diabetes in the community and the implementation of national health insurance coverage for hemodialysis treatment [14]. This condition would suggest a growing number of patients who are susceptible to HCV infection.

Previous studies that investigated local HCV infection outbreaks within dialysis units found common evidence that suggests nosocomial infection with various risk factors related to the dialysis procedure, which were unique to each dialysis unit [2, 15]. Accordingly, the duration of hemodialysis was reported as an independent risk factor for HCV transmission [2, 16–18]. Hemodialysis patients with hepatitis B and HIV infection also have a greater risk of HCV seroconversion [2]. Recent evidence from studies in European countries showed that blood transfusion gave the risk of HCV transmission to less than 1 case/1 million blood transfusion units, related to strict screening assessment [2]. However, data that support this figure in our dialysis unit are not available. Regarding the substantial and growing burden of hepatitis C infection among hemodialysis patients as well as the lack of available data in our hemodialysis unit, we conducted this study to determine the seroconversion and risk factors for HCV infection in our hemodialysis unit.

## 2. Methods

### 2.1. Study Design and Population

This was an analytical cross-sectional study, involving two dialysis units (Sanglah Hospital and one private dialysis unit) in Denpasar, Bali, Indonesia from January 2020 to December 2021. Patient selection and data collection were conducted in June 2022. The selection of the study sample was based on each dialysis unit's patient registry and the data of the eligible sample that was analyzed in this study were collected from patients' charts, both electronic and paper/dialysis records. The inclusion criteria were patients aging ≥ 18 years, undergoing chronic hemodialysis for at least 6 months as scheduled (good adherence), and having complete HCV serology data (initial test and follow-up/screening). Patients who did not have complete HCV serology data, who underwent acute hemodialysis or who were transferred to another hemodialysis unit before 6 months, or who had hepatitis C infection or who tested positive for HCV on the initial screening test before the initiation of dialysis treatment were excluded from this study. This study protocol was reviewed and approved by the Ethical Committee of the Faculty of Medicine, Udayana University, Bali, Indonesia. Written informed consent was not required for this study because data collection was from the medical records.

### 2.2. Clinical Data

Hepatitis C infection was defined by the presence of anti-HCV antibody in the sera detected by enzyme-linked immunosorbent assay. The baseline (the beginning of hemodialysis) and follow-up HCV serology were gathered from medical records. The examination of anti-HCV antibody in patients from the private dialysis unit was conducted in the referral hospital laboratory. Therefore, the anti-HCV antibody examinations of all study population were conducted using the same laboratory kit (Elecsys ® anti-HCV II assay, Roche Diagnostics GmbH, Penzberg, Germany). HCV seroconversion was defined as a change in status from anti-HCV antibody negative at the initiation of dialysis treatment to anti-HCV antibody positive in the latest report. Active HCV infection was verified in patients with positive anti-HCV antibody based on the detectable count of viruses (HCV RNA) on nucleic acid testing and these data were collected from medical records. All of the chronic dialysis patients from both units were regularly screened every 3–6 months for hepatitis C infection by checking anti-HCV antibody. Therefore, we also calculated the annual seroconversion rate within each dialysis unit.

We evaluated several risk factors for HCV transmission from medical records, including age, gender, duration of hemodialysis, type of vascular access, history of blood transfusion, history of surgery, type of dialyzer (reused dialyzer), hepatitis B virus infection, HIV infection, and diabetes mellitus. We evaluated the history of blood transfusion that was received during hemodialysis treatment or on any medical admission that occurred in patient undergoing chronic hemodialysis. Patients with hepatitis B infection were those with positive hepatitis B surface antigen (HBsAg) based on the results of the serology examination.

### 2.3. Statistical Analysis

Gender, type of vascular access, history of blood transfusion, history of surgery, use of dialyzer, hepatitis B virus infection, and diabetes mellitus were presented in frequency and percent, and age and duration of hemodialysis were described by the mean and standard deviation. The Kolmogorov–Smirnoff test was used to evaluate data distribution. We performed bivariate analysis to determine the association between particular risk factors and HCV seroconversion, using chi-square or Fisher exact test. All analyses were two-tailed with a significance level set at 0.05. Factors that showed a significant association with HCV seroconversion in bivariate analysis were entered into multivariate analysis with logistic regression test, to determine if any risk factor was independently associated with HCV infection in a dialysis unit. Data output was expressed as the adjusted odd ratio with a corresponding 95% confidence interval (CI). Statistical analyses were conducted using the software Statistical Package for the Social Sciences version 21.0 (IBM, Armonk, NY, USA).

## 3. Results

There were a total of 396 patients undergoing regular hemodialysis, consisting of 245 patients in the hospital HD unit and 141 patients in the private HD unit, from January 2020 to December 2021. In the hospital HD unit, we excluded 25 patients who were transferred to another dialysis unit less than six months of undergoing hemodialysis in our hospital unit, 8 patients with incomplete HCV serology data, and 2 patients who already had positive anti-HCV before the initiation of dialysis. Thus, 210 patients (85.7%) were enrolled in this study. In the private hemodialysis unit, we excluded 10 patients who were transferred to another dialysis unit less than six months, 12 patients with incomplete HCV serology data, and 1 patient who already had positive anti-HCV before the initiation of dialysis. Thus, 128 patients (84.7%) were enrolled to the study. The total number of patients enrolled in this study was 338 patients.

The mean age of the study population was 51.7 ± 12.3 (20–83) years and 60.9% of our patients were male. Positive anti-HCV antibody was found in 94 patients or 27.8% of the total sample, consisting of 27 patients in the hospital unit (12.9% of the hospital unit population) and 67 patients in the private unit (52.3% of the private unit population). The yearly seroconversion rates in hospital units in 2020 and 2021 were 0.94% and 0.35%, respectively. Meanwhile, the seroconversion rates in private dialysis units were 27.06% in 2020 and 5.55% in 2021. Among patients with positive anti-HCV antibody, the mean duration of dialysis until seroconversion (onset) was 22.6 ± 16.0 (3–72) months. An arteriovenous (AV) fistula was the predominant type (77.2%) of vascular access in our patients and only one patient had undergone surgery that was not related to obtaining vascular access. History of transfusion was found in 44.7% of patients and 56.8% of patients received reused dialyzers. The characteristic data of our patients are presented in Table 1. We found only 43 patients with HCV seropositivity that had HCV RNA data. Among these patients, HCV RNA was detected in 39 patients with a mean of 3.89 × 10^6^ ± 1.48 × 10^7^ (10–9 × 10 [7]), and 4 patients have no detectable HCV RNA.

Older age (≥60 years), male gender, history of blood transfusion, history of surgery, diabetes mellitus, and hepatitis B infection were found to have no significant association with hepatitis C seroconversion on bivariate analysis. We found that vascular access, duration of dialysis, and dialyzer reuse were significantly associated with HCV seroconversion from bivariate analysis (Table 2). However, we found that more patients with positive anti-HCV were having dialysis for less than 5 years compared to those who had dialysis for 5 years or more, which belonged to the private dialysis unit. This figure along with the different practices of dialyzer usage in private dialysis unit (all patients received reused dialyzer), has led to the finding that the type of hemodialysis unit was significantly associated with the risk of hepatitis C infection (OR, 0.134; *p* < 0.001; 95% CI, 0.079–0.229). Accordingly, we conducted a separate bivariate analysis to assess the association of each risk factor with hepatitis C seroconversion within each unit. The evaluation of the hospital HD unit found that the duration of hemodialysis, vascular access, and dialyzer reuse was significantly associated with the risk of HCV infection (Table 3). The analysis of the private HD unit also showed that the duration of hemodialysis and vascular access had a significant association with the risk of HCV infection, along with age and diabetes mellitus (Table 4).

The multivariate analysis of the total study population showed that vascular access, dialyzer reuse, and type of HD unit consistently showed a significant association with HCV infection (Table 5). Although the duration of dialysis was not significantly associated with the risk of HCV infection in a multivariate analysis of the total study population, it showed a significant association with HCV infection in separate analysis, both in hospital (AOR, 4.855; *p* 0.002; 95% CI, 1.762–13.376) and private HD unit patients (AOR, 3.498; *p* 0.001; 95% CI, 1.658–7.379).

## 4. Discussion

The hemodialysis patients in our two dialysis units were predominantly male (60.9%) with a mean age of 51.7 ± 12.3 (20–83) years, which is similar to the findings from other studies in Egypt, Jakarta, and Brazil [8, 18, 19]. We found that the total HCV seroconversion in our dialysis patients was 27.8%. This figure is higher than the reported HCV seropositivity among the general population in Indonesia (1%) [13]. The seroconversion rate in our study was lower than the seroconversion rates reported from other hemodialysis units in Indonesia, that is, 38% in Jakarta and 88% in Surabaya [17, 18]. Hepatitis C seroconversion in our dialysis unit was also lower than the seropositivity reported in studies in Brazil (46.7%), Libya (34.9%), and Egypt (34.8%) [8, 19, 20]. However, the HCV seropositivity in our study was higher than the overall HCV prevalence among Western countries involved in the DOPPS study (9.9%) [2]. Analysis of registry data in several Asia-Pacific countries in 2005 also found a lower frequency of HCV infection among HD patients (7.9%) [21]. Generally, HCV seroconversion among dialysis patients varies considerably across different regions and might reflect how the infection control was carried out in respective dialysis units [2].

Among patients with HCV seropositivity, we found that the mean duration of hemodialysis to hepatitis C seroconversion was 23.4 ± 15.4 (5–72) months. Considering that the median time required for dialysis patients to accumulate anti-HCV antibody is 5 months after the detection of HCV RNA (detectable some days or weeks after infection), the HCV seroconversion observed within 4 months of chronic dialysis initiation could be interpreted as HCV infection that is acquired prior to dialysis [22]. Therefore, all of the seroconversion observed in our study was related to the transmission of infection while undergoing dialysis. Moreover, the majority of the seropositive patients in this study who were tested for HCV RNA were showing detectable viral loads, implying active HCV infection, and capable of transmitting the virus. This condition warrants specific actions to break the chains of infection by investigating the implementation of hygiene standards in the dialysis unit as well as treating the patients with active HCV infection with an antivirus. The recent guideline of the American Association and Study of Liver Disease and Kidney Disease: Improving Global Outcomes (KDIGO) suggests that any dialysis patient who has an active hepatitis C infection and has >1 year of life expectancy should be treated with direct-acting antivirals [23, 24].

The analysis of the associated risk factors for HCV infection in our hemodialysis unit found that dialyzer reuse, vascular access, and type of hemodialysis unit were significantly associated with hepatitis C infection. However, previous studies in European countries have found common evidence that the various risk factors associated with HCV infection were unique to each dialysis unit [2]. This evidence supports the finding of different risk factors in the hospital and private hemodialysis units. The different practices of dialyzer use and time of starting hemodialysis service (the early 1990s in the hospital unit and 2018 in the private unit) might contribute to the differences observed in HCV risk factors in each dialysis unit.

Time on hemodialysis is considered an independent risk factor for HCV seroconversion in dialysis patients [16]. The duration of chronic dialysis treatment may be related to a longer or cumulative exposures to infectious sources that patients who started regular dialysis in previous years had a higher risk of HCV infection, due to less stringent infection control measures [3]. However, the result of our study suggested that those with a duration of dialysis <5 years have a greater risk of HCV seroconversion compared to patients who undergo dialysis ≥5 years and the adjusted association of time on dialysis from both dialysis units with HCV seroconversion was not statistically significant in multivariate analysis. This is possibly explained by the fact that the majority of the patients with seroconversion (71.2%) in our study were those undergoing hemodialysis in the private unit, which started providing dialysis procedures in the last 4 years. Nevertheless, a separate analysis of patients within the private dialysis unit showed that 68.7% of patients with HCV seropositivity had dialysis ≥2 years compared to 39.3% of seronegative patients and this was significantly associated with HCV seroconversion (AOR, 3.498; 95% CI, 1.658–7.379, *p* 0.001). Moreover, a separate analysis on hospital unit patients was showing a statistically significant association with HCV infection using a different time frame of hemodialysis duration (5 years) because the hospital unit started the hemodialysis service in the 1990s. Therefore, it is important to determine an appropriate cut-off point for hemodialysis duration by considering the hemodialysis practices in each unit.

Vascular access (AV fistula) has a significant association with HCV seroconversion in our study (OR, 42.071; 95% CI, 5.757–307.472; *p* value < 0.001). Vascular access sites and extracorporeal blood circuits are indispensable and may add to the risk of acquiring HCV infection while undergoing hemodialysis in a dialysis unit with a high prevalence of HCV [25]. A similar finding by Saxena et al. showed that patients who were dialyzed through an AV fistula have the greatest risk of acquiring HCV infection during dialysis compared to patients who were dialyzed through a polytetrafluoroethylene (PTFE) graft or vascular catheter (temporary and permanent) [25]. Patients with an AV fistula have a higher HCV seroprevalence (61.7%) and 12.3% annual seroconversion (OR, 10.9; 95% CI, 3.2–40.0) compared with a lower prevalence and annual seroconversion rate observed among patients with permanent catheters (12.5% and 0.8%, respectively) [25]. Patients with an AV fistula and a PTFE graft will have regular skin puncture and cannulation to gain vascular access, and thumb pressure for a few minutes is required to stop bleeding from the vascular puncture site at the end of the procedure [25]. Contamination of vascular puncture site could take place at any stage during access handling because the presence of HCV RNA in the hand-washing water of nurses dialyzing HCV-positive and HCV-negative patients has been detected in a clinicovirological study from the Middle East (cross-infection) [26].

Dialyzer reuse showed a significant association with HCV seroconversion in our study (OR, 8.324; 95% CI, 4.319–16.044; *p* value < 0.001). HCV seroconversion was observed in only 8.2% of patients with single-use dialyzers, in contrast to 42.7% of patients with reused dialyzers. However, we observed a disparity regarding the use of a dialyzer between the two dialysis units that were involved in our study. The practice of reprocessing the dialyzer (reuse) is more common in the private dialysis unit, compared to the hospital dialysis unit where all patients undergo dialysis with a single-use dialyzer for the last 6 years. This difference may be responsible for the 71.2% HCV seroconversion in our study in patients who undergo dialysis in the private dialysis unit. Dialyzer reuse has been proposed as a possible risk factor for infection transmission among dialysis patients. Widhani et al. found that dialyzer reuse has a marginal statistically significant association with HCV infection in the dialysis unit and none of the patients with a single-use dialyzer had HCV seroconversion [18]. By contrast, results from multicenter and multinational studies showed that dialyzer reuse was not identified as a risk factor for HCV transmission [2, 27]. Processing a dialyzer several times may cause a microscopic breach in the membrane that may give way to viral transmission [18]. However, according to the guideline by KDIGO, if unavoidable, dialyzer reuse can still be applied to HCV patients, provided that there are implementation and adherence to strict infection control procedures [24]. Therefore, this finding in our study may warrant an investigation of the current infection control procedures in our dialysis unit.

The presence of comorbidities (hepatitis B infection and diabetes mellitus) appeared to have no significant association with HCV seroconversion in our total population of hemodialysis patients. A bivariate analysis on the private dialysis unit, however, showed a significant association between diabetes mellitus and hepatitis C infection among hemodialysis patients, though this association was not significant in multivariate analysis. A recent study has shown a higher risk of acquiring hepatitis C infection in patients with type 2 diabetes compared to the general population, which was considered the result of more frequent exposure to medical intervention and compromised immunity [28]. Results from the DOPPS study found no significant association between diabetes mellitus and HCV positivity among hemodialysis patients [2]. We found 10 patients with hepatitis B infection among hospital unit patients (4.8%) which was lower than the rate of hepatitis B seropositivity found in the previous study in our hospital unit (7.8%) [29]. One of these hepatitis B patients in our study later had HCV seroconversion while undergoing hemodialysis, but the association was not statistically significant.

We found no significant association between a history of blood transfusion (before the screening of anti-HCV antibody) and HCV seroconversion in our patients. This is similar to the result from the study by Widhani et al. and the DOPPS study [2, 18]. A strict screening test before transfusion has accounted for a lower risk of pathogen transmission through blood unit (<1 case per 1 million blood unit) in high-income countries [2]. A previous study in Indonesia evaluated the association between blood transfusion and hepatitis C seropositivity in a large population derived from the Indonesian Basic Health Research and found that this association was not significant statistically [30]. In addition, the widespread use of erythropoietin to treat anemia in chronic kidney disease contributes to fewer transfusion practice and, consequently, decreased risk of HCV transmission [18].

The history of surgery has no significant association with HCV seroconversion in our study. Only one patient in our study had a history of previous surgery which is debridement surgery due to a complication of a diabetic foot, whereas the rest of the patients underwent the same procedures related to vascular access for dialysis. This result agreed with a multicenter study in Egypt and a study in Jakarta, which presumed that medical procedures, including surgery, were not significantly related to HCV infection [8, 18]. However, Alashek et al. found more HCV seroconversion in patients with a history of previous renal transplantation. Hepatitis C infection in these patients might have been transmitted from an infected kidney donor or blood transfused perioperatively. This finding could be related to the shortage of donated kidneys in Libya that compelled many patients to seek a transplant abroad [20].

## 5. Conclusion

Seroconversion of hepatitis C among patients with regular hemodialysis remains high, with various risk factors that were unique to each hemodialysis unit. Factors that were related to the handling of hemodialysis procedures appeared to have an important role in increasing the risk of viral transmission and these considerably vary across different regions. Among HCV seropositive patients, those who had detectable HCV RNA were the potential source of infection within the dialysis unit. Therefore, routine evaluation of infection control measures in the dialysis unit and appropriate antiviral treatment for patients with active HCV infection are needed to prevent further HCV outbreaks within the dialysis unit.

## Figures and Tables

**Table 1:** Characteristic of patients.

Characteristics	Total sample (*n* = 338)
Gender (n, %)	
Male	206 (60.9%)
Female	132 (39.1%)
Age (mean yr, ± SD)	51.7 ± 12.34 (20-83)
Anti-HCV (n, %)	
Reactive	94 (28.2%)
Nonreactive	244 (72.2%)
Diabetes mellitus (n, %)	
Yes	60 (17.8%)
No	278 (82.2%)
Hepatitis B infection (n, %)	
Yes	10 (3.0%)
No	328 (97.0%)
HIV infection	
Yes	0 (0.0%)
No	100 (100%)
Duration of HD (mean month, ± SD)	45.16 ± 29.27 (6-98)
Vascular access (n, %)	
AV fistula	261 (77.2%)
Catheter double lumen	77 (22.8%)
Dialyzer reuse (n, %)	
Yes	192 (56.8%)
No	146 (43.2%)
History of transfusion (n, %)	
Yes	151 (44.7%)
No	187 (55.3%)
History of surgery (n, %)	
Yes	1 (0.3%)
No	337 (99.7%)

**Table 2:** Association of risk factors with HCV infection in hemodialysis patients.

Variable	Anti-HCV (+) (*n* = 94)	Anti-HCV (-) (*n* = 244)	*p* value	Odd ratio	95% CI
Gender					
Male	54 (57.0%)	152 (61.9%)	0.413	0.817	0.504-1.326
Female	40 (43.0%)	92 (38.1%)			
Age					
18-59 yr	65 (69.9%)	186 (74.3%)	0.182	1.431	0.844-2.425
≥60 yr	29 (30.1%)	58 (25.7%)			
Duration of HD					
<5 yr	72 (76.6%)	157 (64.3%)	**0.031**	1.164	0.320-0.951
≥5 yr	22 (23.4%)	87 (35.7%)			
Vascular access					
AV fistula	93 (98.9%)	168 (68.9%)	**<0.001**	42.071	5.757-307.472
Catheter double lumen	1 (1.1%)	76 (31.1%)			
Dialyzer reuse					
Yes	82 (87.2%)	110 (45.1%)	**<0.001**	8.324	4.319-16.044
No	12 (12.8%)	134 (54.9%)			
History of transfusion					
Yes	39 (41.5%)	112 (45.9%)	0.465	0.836	0.516-1.352
No	55 (58.5%)	132 (54.1%)			
History of surgery					
Yes	1 (1.1%)	0 (0%)	0.278^**a**^		
No	93 (98.9%)	244 (100%)			
Diabetes mellitus					
Yes	16 (17.0%)	44 (18.0%)	0.827	1.019	0.497-1.749
No	78 (83.0%)	200 (82.0%)			
Hepatitis B infection					
Yes	1 (1.1%)	9 (3.4%)	0.281	1.256	0.128-2.941
No	93 (98.9%)	235 (96.6%)			
Type of HD unit					
Hospital	27 (28.7%)	183 (75.0%)	**<0.001**	0.134	0.079-0.229
Private	67 (71.2%)	61 (25.0%)			

^∗^cell with value less than 5; Fisher exact test.

**Table 3:** Association of risk factors with HCV infection in hemodialysis patients (hospital unit).

Variable	Anti-HCV (+) (*n* = 27)	Anti-HCV (-) (*n* = 183)	*p* value	Odd ratio	95% CI
Gender					
Male	18 (66.7%)	116 (63.4%)	0.714	1.155	0.491-2.716
Female	9 (33.3%)	67 (36.6%)			
Age					
18-59 yr	20 (74.1%)	135 (73.8%)	0.973	0.984	0.392-2.474
≥60 yr	7 (25.9%)	48 (26.2%)			
Duration of HD					
<5 yr	5 (18.5%)	96 (52.5%)	**0.001**	4.855	1.762-13.376
≥5 yr	22 (81.5%)	87 (47.5%)			
Vascular access					
AV fistula	26 (96.3%)	138 (75.4%)	**0.014**	8.478	1.119-64.261
Catheter double lumen	1 (3.7%)	45 (24.6%)			
Dialyzer reuse					
Yes	15 (55.6%)	49 (26.8%)	**0.002**	3.418	1.496-7.813
No	12 (44.4%)	134 (73.2%)			
History of transfusion					
Yes	12 (44.4%)	84 (45.9%)	0.887	0.943	0.418-2.126
No	15 (55.6%)	99 (54.1%)			
Diabetes mellitus					
Yes	3 (11.1%)	44 (24.0%)	0.132	0.395	0.113-1.374
No	24 (88.9%)	139 (76.0%)			
Hepatitis B infection					
Yes	1 (3.7%)	9 (4.9%)	0.782	0.744	0.90-6.113
No	26 (96.3%)	174 (95.1%)			

**Table 4:** Association of risk factors with HCV infection in hemodialysis patients (private unit).

Variable	Anti-HCV (+) (*n* = 67)	Anti-HCV (-) (*n* = 61)	*p* value	Odd ratio	95% CI
Gender					
Male	36 (53.7%)	36 (59.0%)	0.547	0.806	0.400-1.625
Female	31 (46.3%)	25 (41.0%)			
Age					
18-59 yr	45 (67.2%)	51 (74.3%)	**0.032**	2.493	1.068-5.823
≥60 yr	22 (32.8%)	10 (25.7%)			
Duration of HD					
<2 yr	21 (31.1%)	37 (60.7%)	**0.001**	3.377	1.630-6.995
≥2 yr	46 (68.7%)	24 (39.3%)			
Vascular access					
AV fistula	67 (100%)	30 (49.2%)	**<0.001** ^a^		
Catheter double lumen	0 (0.0%)	31 (50.8%)			
History of transfusion					
Yes	27 (40.3%)	28 (45.9%)	0.522	0.796	0.395-1.604
No	40 (59.7%)	33 (54.1%)			
History of surgery					
Yes	1 (1.5%)	0 (0.0%)	0.338^**a**^		
No	66 (98.5%)	61 (100%)			
Diabetes mellitus					
Yes	13 (19.4%)	0 (0.0%)	**<0.001**		
No	54 (80.6%)	61 (100%)			

^a^cell with value less than 5; Fisher exact test.

**Table 5:** Risk factors for seroconversion—multivariate analysis using logistic regression test.

Variables	AOR	*p* value	95% CI
Duration of HD	1.905	0.301	0.562-6.464
Vascular access	68.876	<0.001	9.061-523.537
Dialyzer reuse	2.336	0.047	1.012-5.388
HD unit	0.146	<0.001	0.071-0.300

## Data Availability

The data used to support the findings of this study are available from the corresponding author upon request.
